# Safety and Preliminary Efficacy of the Acetylcholinesterase Inhibitor Huperzine A as a Treatment for Cocaine Use Disorder

**DOI:** 10.1093/ijnp/pyv098

**Published:** 2015-09-12

**Authors:** Richard De La Garza, Christopher D. Verrico, Thomas F. Newton, James J. Mahoney, Daisy G. Y. Thompson-Lake

**Affiliations:** Baylor College of Medicine, Menninger Department of Psychiatry and Behavioral Sciences, Houston, TX.

**Keywords:** Cocaine, huperzine A, acetylcholinesterase inhibitor, acetylcholine

## Abstract

**Background:**

Cholinergic transmission is altered by drugs of abuse and contributes to psychostimulant reinforcement. In particular, acetylcholinesterase inhibitors, like huperzine A, may be effective as treatments for cocaine use disorder.

**Methods:**

The current report describes results from a double-blind, placebo-controlled study in which participants (n=14–17/group) were randomized to huperzine A (0.4 or 0.8mg) or placebo. Participants received randomized infusions of cocaine (0 and 40mg, IV) on days 1 and 9. On day 10, participants received noncontingent, randomized infusions of cocaine (0 and 20mg, IV) before making 5 choices to receive additional infusions.

**Results:**

Huperzine A was safe and well-tolerated and compared with placebo, treatment with huperzine A did not cause significant changes in any cocaine pharmacokinetic parameters (all *P*>.05). Time-course and peak effects analyses show that treatment with 0.4mg of huperzine A significantly attenuated cocaine-induced increases of “Any Drug Effect,” “High,” “Stimulated,” “Willing to Pay,” and “Bad Effects” (all *P*>.05).

**Conclusions:**

The current study represents a significant contribution to the addiction field since it serves as the first published report on the safety and potential efficacy of huperzine A as a treatment for cocaine use disorder.

## Introduction

A number of medications have been evaluated in clinical trials for cocaine use disorder (Vocci et al., 2005; Haile and Kosten, 2013). Despite these efforts, no medication has gained approval from the Food and Drug Administration for this indication. Although changes in the dopamine (DA) system have been most extensively studied, cholinergic transmission is also altered by drugs of abuse and contributes to psychostimulant reinforcement (Hurd et al., 1990; Mark et al., 1999). DA neurons express multiple types of muscarinic and nicotinic acetylcholine (ACh) receptors, and a dense mingling of dopaminergic and cholinergic neurons in the nucleus accumbens allows coordinated functioning of these neurotransmitter systems (Williams and Adinoff, 2008; Adinoff et al., 2010). Stimulation of ACh inputs can increase DA release in the nucleus accumbens (Berlanga et al., 2003; Mansvelder et al., 2003), and cholinergic interneurons in the nucleus accumbens have been shown to block cocaine-conditioned place preference (Witten et al., 2010).

Importantly, acetylcholinesterase (AChE) inhibitors block cocaine self-administration in monkeys (Wilson and Schuster, 1973), cocaine place preference and locomotor sensitization in mice (Hikida et al., 2003), and reinstatement induced by exposure to methamphetamine in rats (Hiranita et al., 2006). Our laboratory has completed research to determine the effects of the AChE inhibitor rivastigmine on self-reported subjective and reinforcing effects produced by methamphetamine (De La Garza et al., 2008a, 2008b, 2012). In brief, we found that methamphetamine significantly increased several positive subjective effects and rivastigmine significantly reduced these responses. These findings suggest that continued research on this class of compounds is warranted.

Huperzine A (HupA) is a Lycopodium alkaloid isolated from the herb *Huperzia serrata* and is a potent, reversible, and selective AChE inhibitor (Zhang and Tang, 2006). Compared with the AChE inhibitors tacrine, donepezil, and rivastigmine, HupA has better penetration through the blood-brain barrier, higher oral bioavailability, and longer duration of AChE inhibitory action (it is also not a potent butyrylcholinesterase inhibitor) (Wang et al., 2006). Unlike rivastigmine, HupA preferentially inhibits the AChE G4 form, which is the physiologically relevant form at cholinergic synapses, and its inhibition would be expected to prolong the action of ACh (Wang et al., 2006). HupA produces more potent increases in cortical ACh than donepezil and rivastigmine (Liang and Tang, 2004) and more prolonged increases in ACh levels than donepezil, rivastigmine, physostigmine, and other AChE inhibitors (Tang et al., 1989; Zhu and Giacobini, 1995; Liang and Tang, 2004).

In one report, HupA attenuated neuronal degeneration and β-amyloid–induced loss of choline acetyltransferase (ChAT) activity (Wang et al., 2001). This may be important, since long-term cocaine exposure has been associated with reductions in ChAT and AChE inhibitors have been shown to reverse ChAT losses. Beyond ACh, HupA produces a number of effects, both direct and indirect, on other neurotransmitters in the brain (Liang and Tang, 2006; Zhang and Tang, 2006; Qian and Ke, 2014). Of interest, HupA significantly increased prefrontal cortex levels of norepinephrine (42% increase) and DA (112% increase), and these effects were significantly more potent than those observed after donepezil and rivastigmine (Zhu and Giacobini, 1995). HupA reversed memory deficits induced by muscimol, suggesting that some of its effects are mediated through the GABA-A receptor. Antagonism of the GABA-A receptor may be an important mechanism of action for this novel compound, since other compounds acting at this site (and via GABA modulation) have been investigated as potential medications for cocaine use disorder. HupA also acts as a noncompetitive N-methyl-D-aspartate (NMDA) antagonist (Gao et al., 2000). These data indicate that HupA significantly reduced glutamate- and NMDA-induced neurotoxicity (Ved et al., 1997; Gordon et al., 2001). The ability for HupA to antagonize NMDA receptors and potassium currents may contribute to its neuroprotective features. HupA has been evaluated in several trials involving several hundred human patients (Zangara, 2003; Wang et al., 2006; Li et al., 2008; Little et al., 2008) and has been shown to ameliorate deficits in learning and memory (Rafii et al., 2011; Xu et al., 2012; Yang et al., 2013; Xing et al., 2014).

For the current project, we sought to: (1) determine the safety of HupA in cocaine-dependent participants who received cocaine in a laboratory setting, (2) determine the effects of HupA on plasma levels of cocaine and cocaine metabolites, and (3) establish the ability of HupA, as compared with placebo, to attenuate cocaine-induced subjective effects and to reduce reinforcing effects produced by cocaine.

## Methods

### Participants

The current study was a double-blind, placebo-controlled, between-groups evaluation of interactions between intravenous cocaine and oral HupA. Participants were recruited from the Houston metropolitan area through newspaper and radio advertisements. The study was approved by the Baylor College of Medicine and Michael E. DeBakey Veterans Association Medical Center (MEDVAMC) Institutional Review Boards. All participants completed an initial telephone screen to assess basic eligibility. Candidates were then invited to complete an in-person assessment at the Research Commons of the MEDVAMC. During the in-person interview, candidates received an explanation of the study purpose and requirements and were allowed to review, inquire about, and sign the informed consent.

Eligible individuals were required to be between 18 and 55 years of age, provide at least one urine specimen that was positive for cocaine within the 2 weeks prior to study enrollment, meet DSM-5 criteria for cocaine use disorder, and were experienced using cocaine via the smoked or intravenous route. Participants were excluded for any current psychiatric or medical illness, serious neurological or seizure disorder, use of any psychoactive medication, and drug or alcohol use disorders excluding cocaine and nicotine. Women were classified as ineligible for the study if they were pregnant, breast feeding, or not using a reliable form of birth control. Participants were compensated with a $40 gift card for completing the in-person screen and an additional $550 if they completed the inpatient phase of the protocol.

### Drugs

HupA was purchased from Biomedisyn Corporation via Dr. Frank Volvovitz. For this study, BCM submitted an IND that cross-referenced the IND for HupA held by Biomedisyn. In humans, HupA reaches peak concentrations in approximately 60 minutes and T½ was calculated to be approximately 4 hours (Qian et al., 1995; Li et al., 2007). On the basis of this information, treatment with HupA for several days for the current study was deemed adequate to achieve steady-state levels. The doses for HupA were selected on the basis of several individual studies and also a meta-analysis of clinical trials showing that these doses have beneficial effects on improving cognitive function and global clinical assessment in patients with Alzheimer’s disease (Yang et al., 2013).

HupA and matching placebo were encapsulated by Greenpark Pharmacy (Houston, TX). Human use cocaine HCl was provided by Research Triangle Institute International (Research Triangle Park, NC).

### Procedures

All study visits took place at the MEDVAMC Research Commons. Initial screening measures included a medical and drug use history, electrocardiogram, and vital signs, which were conducted by trained research staff. Eligible participants were admitted to the Research Commons as inpatients and then randomized into 1 of 3 groups: HupA (0.4 or 0.8mg) or placebo. It is important to note that the placebo cohort described in this report is also used as a comparison group in a companion paper that describes outcomes for 2 additional, but distinct, test groups (Rivastigmine 3 and 6mg; De La Garza et al., unpublished observations).

#### Pre-randomization (Day 1)

Subjects received double-blind infusions of saline or cocaine (40mg, IV) at 10 am and 2 pm on day 1 prior to randomization to study drug. Specifically, each participant received cocaine in one session and placebo in the other session on day 1 (and on day 9 as described below). Saline and cocaine were administered by IV push over 2 minutes by a study physician. Heart rate and blood pressure were recorded at 15 minutes prior (T=-15 minutes), and at 5, 10, 15, 20, 30, 45, 60, 90, and 120 minutes following each infusion. To assess subjective effects, visual analogue scales (VAS) were completed before and after each infusion at the same time-points specified for cardiovascular measures. VAS data were collected for ratings of “Any Drug Effect,” “Stimulated,” “Pay,” (ie, “How much are you willing to pay for the infusion you just received-in dollars?),” “Good Effects,” “Like,” “Desire Cocaine,” “High,” “Likely to Use if Given Access,” “Anxious,” “Bad Effects,” and “Depressed.” These scales ranged from 0 (no effect) to 100 (greatest effect ever).

#### Study Medication Randomization and Dose Escalation

On days 2 to 10, HupA or placebo was administered orally twice daily at 8:00 am and 6:00 pm according to the schedule below. To assure the safety of subjects, a gradual dose escalation took place. The 0.4-mg HupA group received 0.2mg only in the evening of day 1, 0.2mg in the morning and evening of days 2 to 9, and 0.2mg in the morning of day 10. The 0.8-mg HupA group received 0.2mg only in the evening of day 1, 0.2mg in the morning and evening of days 2 to 5, 0.4mg in the morning and evening of days 6 to 9, and 0.4mg in the morning of day 10.

#### Post-randomization (Day 9)

The procedures on this day were identical to those described above for day 1, though these were considered post-randomization responses and these data were used to evaluate medication effects.

#### Self-Administration Session (Day 10)

On day 10, participants completed 2 self-administration/choice sessions, one at 10:00 am and the second at 1:00 pm. At the start of each session, participants were provided with $25 from their study earnings (five $5 bills, one for each choice opportunity). Participants were first given a non-contingent infusion of saline or cocaine (20mg, IV) as a sample of the dose that was available during that session. Subsequently, participants were given 5 opportunities, at 15-minute intervals, to purchase additional infusions (the same as the sample dose) or to keep $5 for that choice opportunity. Infusion choices were performed by the patient using a patient-controlled analgesia pump, while choices for money were indicated verbally to the research coordinator. Patient-controlled analgesia pump infusions occurred over 2 minutes followed by a 13-minute time-out period. As such, selections were made at 15-minute intervals. Participants received infusions immediately after indicating their choice, providing vital signs remain within preset limits.

For this session, heart rate and blood pressure were recorded at 15 minutes prior (T=-15min) and at 5 and 10 minutes following each infusion or choice opportunity. To assess subjective effects, VAS forms were completed before and after each choice opportunity at the same time-points specified for cardiovascular measures.

#### Study Conclusion

Study drug was discontinued after the morning session on day 10 (ie, medication was not administered in the evening on this day). Subjects were then monitored overnight and discharged the following day if they were deemed medically stable.

### Safety

Adverse events (AEs) were summarized by Medical Dictionary for Drug Regulatory Affairs system organ class, preferred term, and observation period (placebo-only period, placebo-cocaine period, and treatment-cocaine period) for overall incidence, incidence by severity, incidence by relationship to study drug, and incidence by relationship to cocaine.

A physician was present during all cocaine infusion sessions to carefully monitor heart rate, blood pressure, and ECG wave form. Stopping rules were in place to halt cocaine administration under conditions in which cardiovascular indices exceeded preset values, although cocaine was not withheld for any subject during any experimental sessions described in this report.

### Pharmacokinetic Analyses

The interaction effects of HupA on the pharmacokinetics (PK) of cocaine and its major metabolites were assessed by measuring plasma PK parameters following IV infusion of 40mg of cocaine during treatment with placebo (on day 1) vs treatment with HupA or placebo (day 9). Blood samples were collected at -15, 2, 5, 15, 30, 60, 90, 120, 150, and 180 minutes following dosing of 40mg of cocaine. Blood was collected into Vacutainer tubes containing potassium oxalate and sodium fluoride to inhibit cocaine hydrolysis by plasma cholinesterases. After separation of plasma by routine centrifugation, samples were frozen at -70°C until analysis.

Plasma levels of cocaine and metabolites were assayed by liquid chromatography-tandem mass spectrometry with a lower limit of quantitation of 2.5ng/mL at the University of Utah Center for Human Toxicology under the direction of David E. Moody, PhD. Cocaine, benzoylecgonine, and ecgonine methyl ester were measured (norcocaine was not detectable in any samples). Bioanalysis was performed using validated liquid chromatography-tandem mass spectrometry methodology with a linear quantitation range of 0.5 to 500ng/mL.

### Statistical Analyses

Data were analyzed using StatView 5.0 (SAS Institute Inc., Cary, NC). For all measures, statistical significance was set at *P*<.05. All data are presented as mean ± SEM.

Demographic information and drug use data were analyzed using appropriate parametric or nonparametric tests. Safety assessments included the number, type, and severity of AEs. AE data were analyzed by ANOVA as a function of HupA dose (0, 0.4, or 0.8mg). ECG data were also recorded during all infusion sessions as part of safety assessments, but there were no differences among treatment groups and those data are not shown.

PK data were calculated based on the baseline (pre-randomization, day 1) vs post-randomization cocaine infusion (40mg, IV, on day 9). Plasma concentration-time profiles were analyzed to obtain PK parameter estimates of cocaine, including area under the time-curve (AUC), maximum concentration, and time to maximum concentration. Data were analyzed using ANOVA with day (Pre-Rand vs Post-Rand) and HupA dose (0, 0.4, or 0.8mg) as factors.

To determine treatment effects for cardiovascular and subjective effects, data from post-randomization (day 9) outcomes were used. Effects across the time-course were calculated using AUC and analyzed using ANOVA with HupA dose (0, 0.4, or 0.8mg) and cocaine dose (0 or 40mg) as factors. Exploratory pair-wise comparisons within the 40-mg cocaine dose were conducted using the Holm-Sidak method.

For the self-administration day, cardiovascular measures and subjective effects data following the non-contingent infusion were averaged given that there were only 2 time-points (5 and 10 minutes). These data, as well as number of choices for infusions, were analyzed using ANOVA with HupA dose (0, 0.4, or 0.8mg) and cocaine dose (0 or 20mg) as factors. Exploratory pair-wise comparisons within the 20-mg cocaine dose were conducted using the Holm-Sidak method.

## Results

### Participants

Eligible volunteers were randomized to placebo (n*=*16), HupA 0.4mg (n*=*17), and HupA 0.8mg (n*=*14). On average, participants were African American males who were approximately 42 years of age. The majority of participants smoked approximately 2g of cocaine per day on the majority of days (>15) each month. An overwhelming majority of participants (>85%) also drank alcohol and smoked cigarettes. Analyses revealed no significant differences among groups for demographic or drug use variables (Table 1).

**Table 1.** Demographic and Drug Use Characteristics

	**Placebo** (n=16)	**Hup A 0.4** (n=17)	**Hup A 0.8** (n=14)
Gender			
Male	14	13	10
Female	2	4	4
Race/ethnicity			
African American	12	14	8
Caucasian	0	2	5
Hispanic	1	1	1
Other	3	0	0
Age	40.4±2.0	44.5±1.4	41.5±2.3
Education (years)	12.1±0.3	12.7±0.5	12.6±0.6
Cocaine use			
Years	15.9±1.8	17.1±1.9	15.5±2.2
Recent^*a*^	15.4±1.7	17.7±2.2	17.1±2.9
grams/day	1.8±0.3	2.0±0.3	2.1±0.4
Nicotine use	88%	88%	100%
Years	17.6±2.1	24.3±2.0	17.7±1.9
Cigarettes/day	10.4±2.0	12.7±2.4	10.9±1.7
Alcohol use	94%	88%	86%
Years	19.1±2.2	21.1±2.4	21.2±2.7
Recent	9.5±2.1	6.5±1.9	13.3±2.7

Values represent mean±SEM.

^*a*^Number represents days used in the past 30 days.

### AEs

The majority of AEs were mild or moderate (eg, headache, stomachache), and there were no AEs reported as severe or very severe/life-threatening. In addition, there were no serious AEs and no discontinuations from the study due to AEs. Analyses showed no statistical differences in the number or severity of AEs among treatment groups (F_2,119_=.82, *P*=.44).

### PKs

Summary statistics of key PK parameters for cocaine determined from plasma samples collected following IV administration of 40mg of cocaine on days 1 and 9 are shown in Table 2. In comparison with placebo, treatment with HupA did not cause significant changes in any cocaine PK parameters (all *P*>.05).

**Table 2.** Pharmacokinetic Parameters for Cocaine and Metabolites Following Cocaine (40mg, IV) vs Saline on Days 1 and 9

		Study Day 1			Study Day 9		
		Placebo	Hup 0.4	Hup 0.8	Placebo	Hup 0.4	Hup 0.8
Cocaine	AUC	15965.1±5130.1	17920.9±4683.1	23401.4±5310.1	18143.2±5130.1	24286.5±5310.1	34920.9±5735.6
	C_MAX_	338.9±708.1	1140.5±667.6	772.8±756.9	474.1±731.3	2598.9±708.1	1498.1±756.9
	T_MAX_	4.9±1.9	3.8±1.8	5.4±2.1	6.2±2.0	2.2±1.9	8.9±2.1
BE	AUC	20587.9±1854.2	19631.6±1692.7	24985.6±1919.3	20102.5±1854.2	21980.9±1919.3	22528.5±2073.1
	C_MAX_	214.8±153.1	287.7±144.3	267.7±163.7	222.8±158.1	619.2±153.1	307.8±163.7
	T_MAX_	99.4±9.1	82.6±8.5	84.6±9.7	93.0±9.4	84.8±9.1	90.1±9.7
EME	AUC	4063.2±535.8	2276.7±489.2	2759.9±554.7	2460.8±535.9	2495.1±554.7	2473.3±599.1
	C_MAX_	43.7±9.7	32.4±9.1	31.6±10.3	30.5±9.9	44.8±9.7	34.4±10.3
	T_MAX_	99.5±9.9	98.4±9.4	89.3±10.7	90.1±10.3	57.0±9.9	73.1±10.7

All data reflect mean±S.E.M.; C_MAX_ data reflect maximum concentration in ng/ml; T_MAX_ data reflect time to maximum concentration in minutes

BE=benzoylecgonine, EME=Ecognine Methylester

### Post-randomization Cardiovascular Responses Day 9

**Figure 1.**
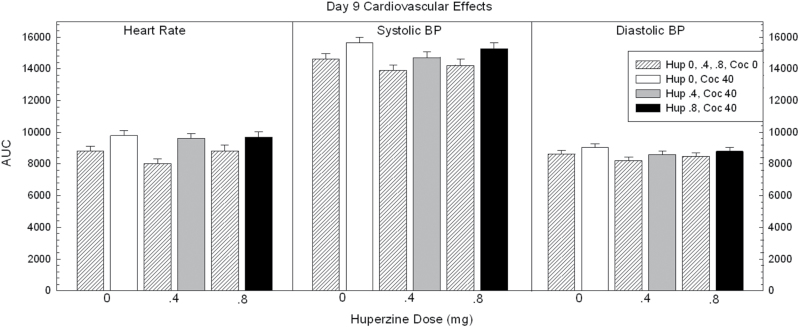
Area under the curve (AUC) data for cardiovascular responses on day 9 in participants treated with huperzine A (HupA) (0, 0.4, and 0.8mg) and cocaine (0 and 40mg). Values represent mean±SEM.

Calculated AUC data for heart rate and systolic and diastolic blood pressure are shown in Figure 1.

For heart rate, ANOVA of the AUC revealed no main effect of HupA (F_2,88_=1.46, *P*=.24), a significant effect for cocaine (F_1,88_=18.8, *P*<.0001), but no significant HupA × cocaine interaction (F_2,88_=.74, *P*=.48). Exploratory pair-wise comparisons within the 40-mg cocaine dose showed no significant differences between placebo vs HupA 0.4mg (t=.42, *P*=.67) or placebo vs HupA 0.8mg (t=.22, *P*=.82).

For systolic blood pressure, ANOVA of the AUC revealed no main effect of HupA (F_2,88_=2.70, *P*=.07), a significant effect for cocaine (F_1,88_=10.6, *P*<.0001), but no significant HupA × cocaine interaction (F_2,88_=.07, *P*=.93). Exploratory pair-wise comparisons within the 40-mg cocaine dose showed no significant differences between placebo vs HupA 0.4mg (t=1.87, *P*=.06) or placebo vs HupA 0.8mg (t=.75, *P*=.46).

For diastolic blood pressure, ANOVA of the AUC revealed no main effect of HupA (F_2,88_=1.68, *P*=.19), no significant effect for cocaine (F_1,88_=3.66, *P*=.06), and no significant HupA × cocaine interaction (F_2,88_=.02, *P*=.98). Exploratory pair-wise comparisons within the 40-mg cocaine dose were not conducted.

### Post-randomization Subjective Effects Day 9

**Figure 2.**
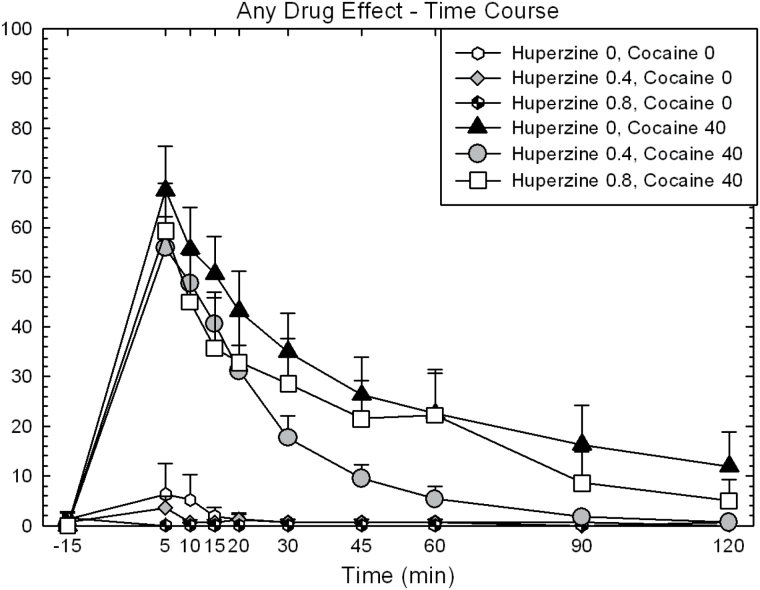
Time-course data for “Any Drug Effect” on day 9 in participants treated with huperzine A (HupA) (0, 0.4, and 0.8mg) and cocaine (0 and 40mg). Values represent mean±SEM. The symbols shown in this figure are identical for those used in Figures 3 and 4.

**Figure 3.**
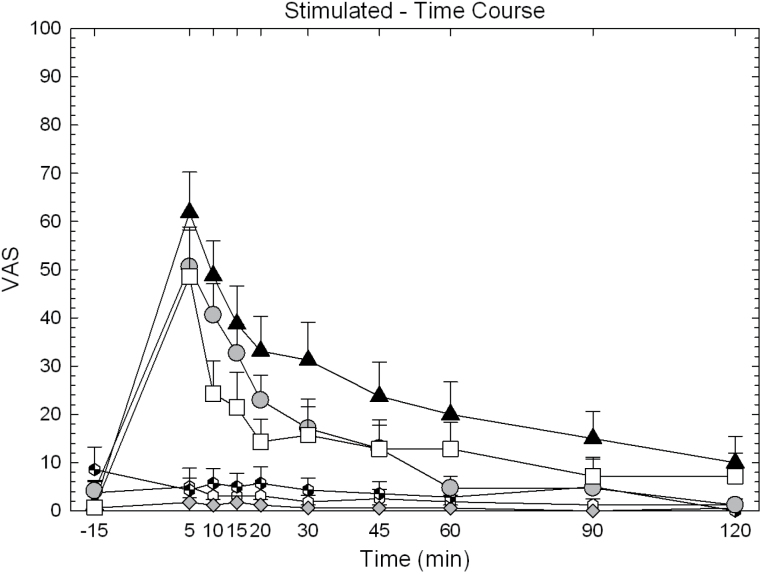
Time-course data for “Stimulated” on day 9 in participants treated with huperzine A (HupA) (0, 0.4, and 0.8mg) and cocaine (0 and 40mg). Values represent mean±SEM.

**Figure 4.**
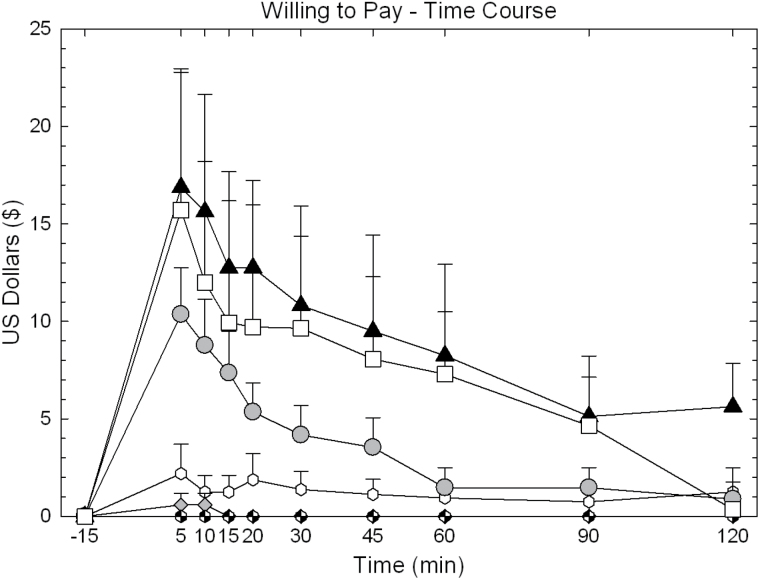
Time-course data for “Willing to Pay” on day 9 in participants treated with huperzine A (HupA) (0, 0.4, and 0.8mg) and cocaine (0 and 40mg). Values represent mean±SEM.

**Figure 5.**
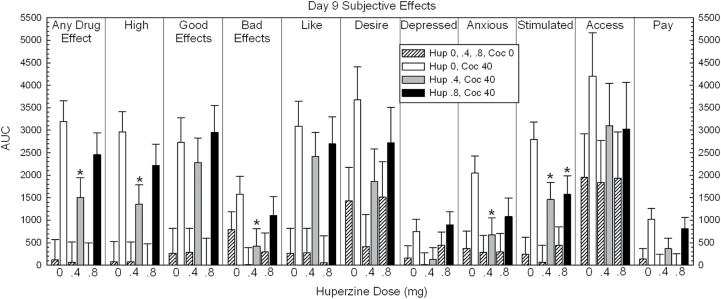
Area under the curve (AUC) data for all subjective effects on day 9 in participants treated with huperzine A (HupA) (0, 0.4, and 0.8mg) and cocaine (0 and 40mg). Values represent mean±SEM. *P<.05 vs placebo.

Full time-course data for “Any Drug Effect,” “Stimulated,” and “Willing to Pay” are shown for illustrative purposes in Figures 2, 3 and 4. Calculated AUC data are shown in Figure 5.

For “Any Drug Effect,” ANOVA of the AUC revealed no main effect of HupA (F_2,88_=1.86, *P*=.16), a significant effect for cocaine (F_1,88_=37.52, *P*<.0001), but no significant HupA × cocaine interaction (F_2,88_=1.70, *P*=.19). Exploratory pair-wise comparisons within the 40-mg cocaine dose showed a significant reduction of effect for HupA 0.4mg vs placebo (t=2.66, *P*=.009), but not HupA 0.8mg vs placebo (t=1.11, *P*=.27).

For “Stimulated,” ANOVA of the AUC revealed no main effect of HupA (F_2,88_=2.00, *P*=.14), a significant effect for cocaine (F_1,88_=28.19, *P*<.0001), but no significant HupA × cocaine interaction (F_2,88_=1.85, *P*=.16). Exploratory pair-wise comparisons within the 40-mg cocaine dose showed a significant reduction of effect for HupA 0.4mg vs placebo (t=2.47, *P*=.015) and a significant reduction of effect for HupA 0.8mg vs placebo (t=2.15, *P*=.034).

For “Willing to Pay,” ANOVA of the AUC revealed no main effect of HupA (F_2,88_=1.38, *P*=.26), a significant effect for cocaine (F_1,88_=11.94, *P*<.0001), but no significant HupA × cocaine interaction (F_2,88_=.72, *P*=.49). Exploratory pair-wise comparisons within the 40-mg cocaine dose showed a significant reduction of effect for HupA 0.4mg vs placebo (t=1.96, *P*=.05), but not HupA 0.8mg vs placebo (t=.60, *P*=.55).

Significant effects were also shown between placebo vs HupA 0.4mg within the 40-mg dose for several other subjective effects, including “High,” “Bad Effects,” and “Anxious” (statistical outcomes not described).

### Cardiovascular Responses Day 10

**Figure 6.**
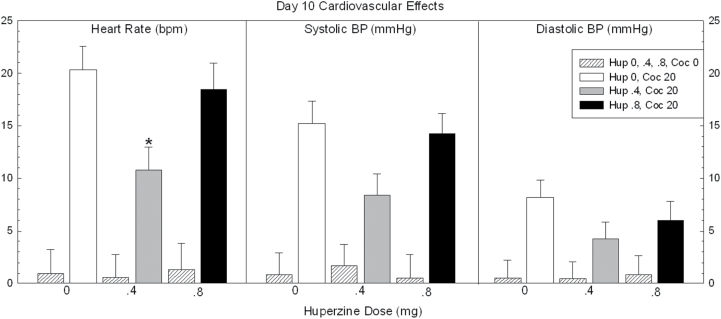
Cardiovascular responses on day 10 in participants treated with huperzine A (HupA) (0, 0.4, and 0.8mg) and cocaine (0 and 20mg). Values represent mean±SEM. * *P*<.05 vs placebo.

Data for heart rate and systolic and diastolic blood pressure are shown in Figure 6. For heart rate, ANOVA revealed no main effect of HupA (F_2,44_=2.38, *P*=.10), a significant effect for cocaine (F_1,44_=70.0, *P*<.0001), but no significant HupA × cocaine interaction (F_2,44_=2.98, *P*=.06). Exploratory pair-wise comparisons within the 20-mg cocaine dose showed a significant reduction of effect for HupA 0.4mg vs placebo (t=3.1, *P*=.01), but not HupA 0.8mg vs placebo (t=.54, *P*=.59).

For systolic blood pressure, ANOVA revealed no main effect of HupA (F_2,44_=1.69, *P*=.19), a significant effect for cocaine (F_1,44_=81.3, *P*<.0001), but no significant HupA × cocaine interaction (F_2,44_=2.78, *P*=.07). Exploratory pair-wise comparisons within the 20-mg cocaine dose showed no significant effect for HupA 0.4mg vs placebo (t=2.3, *P*=.07) nor HupA 0.8mg vs placebo (t=.30, *P*=.76).

For diastolic blood pressure, ANOVA revealed no main effect of HupA (F_2,44_=.89, *P*=.42), a significant effect for cocaine (F_1,44_=18.6, *P*<.0001), but no significant HupA × cocaine interaction (F_2,44_=.73, *P*=.49). Exploratory pair-wise comparisons within the 20mg cocaine dose showed no significant effect for HupA 0.4mg vs placebo (t=1.7, *P*=.26) nor HupA 0.8mg vs placebo (t=.87, *P*=.63).

### Subjective Effects and Choice Data Day 10

**Figure 7.**
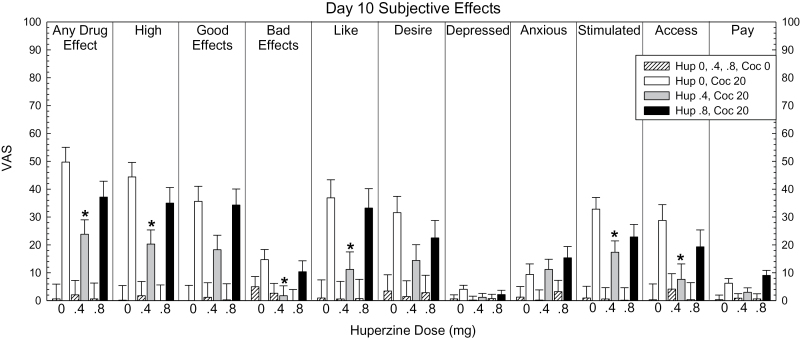
Subjective effects data for all visual analogue scale (VAS) adjectives on day 10 in participants treated with huperzine A (HupA) (0, 0.4, and 0.8mg) and cocaine (0 and 20mg). Values represent mean±SEM. * *P*<.05 vs placebo.

Subjective effects data for all VAS adjectives are shown in Figure 7. For “Any Drug Effect,” ANOVA revealed no main effect of HupA (F_2,44_=2.68, *P*=.08), a significant effect for cocaine (F_1,44_=64.62, *P*<.0001), and a significant HupA × cocaine interaction (F_2,44_=3.41, *P*=.042). Pair-wise comparisons within the 20-mg cocaine dose showed a significant reduction of effect for HupA 0.4mg vs placebo (t=3.48, *P*=.0002), but not HupA 0.8mg vs placebo (t=1.61, *P*=.11).

For “High,” ANOVA revealed no main effect of HupA (F_2,44_=2.45, *P*=.098), a significant effect for cocaine (F_1,44_=55.21, *P*<.0001), and a significant HupA × cocaine interaction (F_2,44_=3.14, *P*=.05). Pair-wise comparisons within the 20-mg cocaine dose showed a significant reduction of effect for HupA 0.4mg vs placebo (t=3.31, *P*=.0004), but not HupA 0.8mg vs placebo (t=1.23, *P*=.22).

For Likely to Use if Given Access (“Access”), ANOVA revealed no main effect of HupA (F_2,44_=1.37, *P*=.27), a significant effect for cocaine (F_1,44_=11.19, *P*<.0001), but no significant HupA × cocaine interaction (F_2,44_=2.22, *P*=.12). Exploratory pair-wise comparisons within the 20-mg cocaine dose showed a significant reduction of effect for HupA 0.4mg vs placebo (t=2.66, *P*=.027), but not HupA 0.8mg vs placebo (t=1.14, *P*=.26).

Significant effects were shown between placebo vs HupA 0.4mg within the 20-mg dose for several other subjective effects, including “Like,” “Stimulated,” and “Bad Effects” (statistical outcomes not described).

For “Choices” (data not shown), ANOVA revealed no main effect of HupA (F_2,44_=2.48, *P*=.09), a significant effect for cocaine (F_1,44_=44.57, *P*<.0001), but no significant HupA × cocaine interaction (F_2,44_=2.48, *P*=.09). Exploratory pair-wise comparisons within the 20-mg cocaine dose showed no significant difference between HupA 0.4mg vs placebo (t=1.99, *P*=.097) nor HupA 0.8mg vs placebo (t=1.15, *P*=.25).

## Discussion

The current report provides for the first time preliminary data for the potential efficacy of HupA as a treatment for cocaine use disorder. Overall, HupA was well-tolerated, did not alter cocaine PKs, and notably decreased several subjective effects produced by cocaine at 2 doses. These findings correspond well with our own research investigating the AChE inhibitor rivastigmine for methamphetamine use disorder (De La Garza et al., 2008a, 2008b, 2012) and are in agreement with numerous preclinical reports showing the importance of ACh in modulating DA function in the nucleus accumbens and cocaine-induced behaviors. Despite these findings, clinical research with the AChE inhibitor, donepezil, has not been positive. In a 10-week outpatient clinical trial of cocaine-dependent subjects, craving and self-reported cocaine use were statistically similar in participants treated with donepezil (10mg) and placebo (Winhusen et al., 2005). In an inpatient laboratory study, treatment with donepezil (5mg) for 3 days increased ratings of “any” and “good” drug effect produced by low-dose cocaine, but did not modify responses to high-dose cocaine (Grasing et al., 2010). Failed outcomes with donepzil, as compared with HupA, may be easy to explain given a wealth of published data. Namely, compared with donepezil, HupA has better penetration through the blood-brain barrier, higher oral bioavailability, and longer duration of AChE inhibitory action (Wang et al., 2006). In addition, HupA produces more potent increases in cortical ACh than donepezil (Liang and Tang, 2004) and more prolonged increases in ACh levels than donepezil (Tang et al., 1989; Zhu and Giacobini, 1995; Liang and Tang, 2004). Perhaps most important, as mentioned above, HupA has effects on other neurotransmitter systems (eg, glutamate) that may contribute to its apparent efficacy for cocaine use disorder as compared with donepezil.

Of interest, the observed reductions in the current study were reported at the 0.4-mg but not 0.8-mg dose level. There are several possible explanations for this outcome, one of which is that these results reflect an inverted-U dose effect (commonly observed elsewhere in pharmacology, eg, DA effects on cognitive function) in which low doses may be ineffective, moderate doses are efficacious, and high doses are not effective because of a saturation effect that may lead to behavioral impairments that mask any effects seen at moderate dose levels (eg, Cools and D’Esposito, 2011). It is also important to mention that there is some evidence that anticholinergic drugs may have beneficial effects for cocaine use disorder, specifically biperiden, a cholinergic antagonist, reduced cocaine craving in an outpatient clinical trial (Dieckmann et al., 2014). These outcomes highlight the possibility that the effects produced by HupA in the current study may not be the result of an increase ACh but also the actions of HupA on other neurotransmitters. The complex effects of nicotinic vs muscarinc agonists and antagonists as they pertain to cocaine addiction have been summarized previously (Adinoff et al., 2010).

Despite reducing several positive subjective effects, HupA produced only moderate, nonsignificant reductions in choices for cocaine in the laboratory. Self-administration is considered a key proxy for drug use (Haney and Spealman, 2008) and the absence of significant effects in the current study may have been the result of the relatively short treatment duration (10 days). On this basis, the absence of impact on cocaine choice reduces confidence that this represents a useful treatment for cocaine use disorder, though this remains to be known. It is possible that longer treatment exposure (8–12 weeks), as is common for outpatient clinical trials might accentuate the ability for HupA to reduce cocaine use in this population.

Despite the positive outcomes reported, there were a few notable limitations of the current study, including: (1) many statistical tests on effects of the medication conditions were conducted, but only a few were significant, which raises the possibility that the findings represent capitalization on chance and Type I error; (2) some significant effects occurred with the lower, rather than the higher, dose of HupA; (3) the effects appear to be modest and confined to the subjective effects; and (4) the relatively small sample size for each group may limit the conclusions that can be drawn from the current data.

Like other AChE inhibitors, HupA has been most commonly evaluated in patients with Alzheimer’s disease (Yang et al., 2013; Qian and Ke, 2014) with specific focus on its ability to improve cognition (Rafii et al., 2011; Xu et al., 2012). This may be particularly relevant for addiction, since long-term cocaine use is a risk factor for impaired neurocognition (Sofuoglu, 2010; Spronk et al., 2013). One strategy for addressing the issue of poor treatment outcomes (ie, treatment retention) in cocaine-dependent individuals is to identify pharmacologic agents that can reverse frontal/executive dysfunction (Sofuoglu et al., 2013), and future studies should evaluate the effects produced by HupA on cognitive function itself, and this medication might be combined with cognitive behavioral therapy in future studies.

## Statement of Interest

None.
